# Surgical instrument wrap: a pilot recycling initiative

**DOI:** 10.1007/s11845-023-03491-7

**Published:** 2023-09-21

**Authors:** David James Rooney, Laura Linehan, Cathy Burke

**Affiliations:** 1 Department of Obstetrics and Gynaecology, University Hospital Waterford, Waterford, Ireland; 2 Department of Obstetrics and Gynaecology, Cork University Maternity Hospital, Cork, Ireland; 3 University College Cork, Cork, Ireland

**Keywords:** Instrument, Operating, Recycling, Surgical, Theatre, Wrap

## Abstract

**Background:**

Seven per cent of general waste and 20% of healthcare risk waste produced in acute hospitals in Ireland comes from operating theatres. Surgical wrap comprises 11% of operating theatre waste.

**Aims:**

The primary aim of this study was to pilot the implementation of a recycling initiative for surgical instrument set wrap in an operating theatre in Ireland. Secondary aims included quantification of the surgical wrap diverted from general waste to recycling streams over a 5-week period and estimation of the annual carbon emissions associated with gynaecology surgical wrap use in Cork.

**Methods:**

The amount of polypropylene surgical wrap generated by a single gynaecology theatre at Cork University Maternity Hospital was prospectively quantified from 24/1/22 to 1/3/22. At the end of the study period, individual sheets of polypropylene wrap were counted and dimensions were measured to calculate the total surface area of surgical wrap saved for recycling.

**Results:**

A total of 66 surgeries were performed over the 5-week study period. Two hundred twenty-one individual sheets of surgical wrap were collected, equating to 282.1 m^2^ of polypropylene wrap. An estimated 11,564 m^2^ of surgical wrap could be recycled annually from the gynaecology theatre service in Cork with an associated annual carbon emissions equivalent of at least 2.2 tonnes of CO_2_.

**Conclusion:**

Diversion of surgical wrap from general waste and clinical waste streams to the recycling stream is achievable in every operating theatre. Small changes to operating theatre waste disposal practices have the potential to yield significant reductions to theatre waste outputs and to hospital carbon emissions.

## Introduction

The healthcare sector generates millions of tonnes of waste worldwide each year [1], thus contributing significantly to global warming and climate breakdown. Approximately 85% of hospital-generated waste is general waste which is non-hazardous, and much of this is suitable for recycling [1]. It is estimated that 17,000 tonnes of such non-hazardous waste is generated by hospitals in Ireland each year [2]. Ireland’s Environmental Protection Agency Green Healthcare Programme (GHCP) has reported that 32% of materials found in non-hazardous waste bins in Irish hospitals are recyclable materials [2]. Recyclable materials often get mixed with clinical risk waste at the point of generation, resulting in high environmental and cost implications [1]. Ultimately, recyclable materials such as plastics can end up in landfill or waste incinerators, with resultant environmental consequences.

**Fig. 1:**
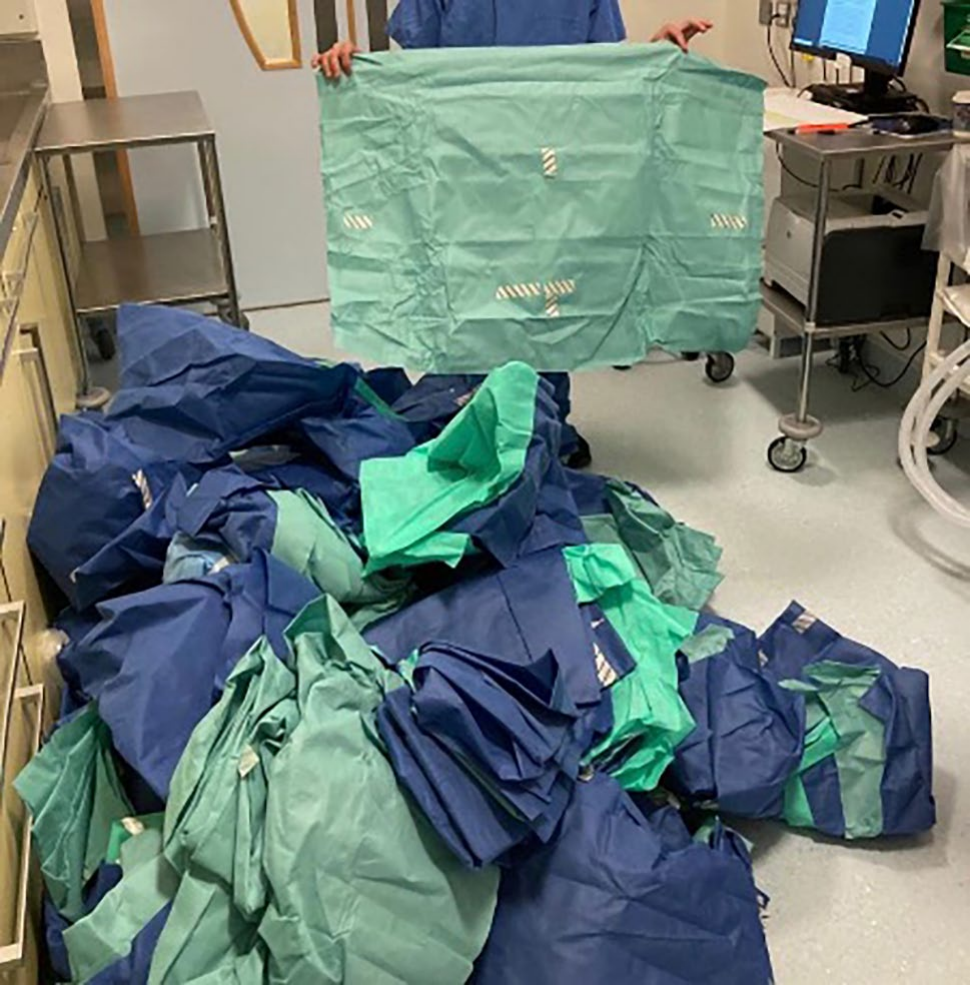
Polypropylene surgical wrap

Seven percent of general waste and 20% of healthcare risk waste produced in acute hospitals in Ireland comes from operating theatres [3, 4]. A key component of theatre waste reduction is the segregation of recyclables from both non-risk and healthcare risk waste [4]. Surgical set wrap, which surrounds sterile surgical instrument sets, is a recyclable plastic material made from polypropylene, itself manufactured from fossil fuels (Fig. 1). At Cork University Maternity Hospital (CUMH), surgical sets are surrounded by two layers of surgical wrap—an external layer of blue wrap and an inner layer of green wrap (Fig. 1). These layers of wrap are bulky in nature, rapidly fill waste bags and comprise 11% of all waste generated in the operating theatre [4, 5]. Segregation of this material for recycling can therefore reduce the volume of both non-risk and healthcare risk waste generated in hospital theatres, and reduce the carbon footprint of hospitals.

There are cost benefits to increasing recycling rates in operating theatres. It is three times less costly to dispose of materials as mixed dry recyclables rather than as general waste [3]. The GHCP has estimated that savings of between €380,000 and €550,000 per annum could be made in acute Irish hospitals nationally by ensuring that recyclables are kept segregated from general waste [3].

The primary aim of this study was to pilot the implementation of a recycling initiative for surgical instrument set wrap in an operating theatre in Ireland. Secondary aims included measurement of the quantity and surface area of surgical wrap which could be diverted from general waste to recycling streams over a 5-week period, as well as estimation of the annual carbon emissions associated with gynaecology surgical wrap use in Cork, some of which could be mitigated as a result of increased diversion to recycling.

## Methods

A surgical wrap recycling initiative was piloted in a single gynaecology theatre at CUMH over a 5-week period from 24/1/22 to 1/3/22, inclusive.

Consultation with local stakeholders was conducted prior to implementation of the initiative. To ensure appropriate waste management procedures could be implemented, the authors liaised with hospital waste management services to agree pre-specified recycling procedures. It was agreed that polypropylene wrap would be eligible for recycling, with the stipulations that the material was not contaminated with potentially hazardous infectious material and was baled prior to recycling. Following consultation with hospital management, funding was obtained to allow for rental of appropriate baling equipment for an initial 3-month period at a cost of €70 per month. There was no charge levied for the waste itself or for onward movement of the baled polypropylene to the recycling centre.

Within the operating theatre, an information session was held by the theatre manager to provide training to theatre staff on the processes associated with the initiative. Theatre nursing staff removed and segregated the surgical wrap from instrument sets at the start of each theatre case, and placed it in a designated container in the set-up room. Theatre porters collected the material and placed it in a separate designated bin in the theatre waste room. This bin was clearly labelled for the purpose of collecting polypropylene surgical wrap solely. At the end of the study period, the individual sheets of polypropylene wrap were counted and dimensions measured to calculate the total surface area of surgical wrap saved for recycling. The surgical wrap sheets were then baled in the waste yard. The hospital’s waste management company collected the baled wrap and sent it for onward recycling in Ireland into polypropylene pellets, which are used for the manufacture of plastic buckets in the UK.

The gynaecological procedures undertaken during the study period were reviewed retrospectively to calculate both the number of surgeries and categorisation of surgeries performed, as well as the estimated number of instrument sets used per case. The estimated volume of surgical wrap which could be recycled annually was then calculated. This calculation was based on the number of gynaecological surgeries performed in Cork in 2019, the most recent fully operational year prior to the COVID-19 pandemic. Calculations assumed a similar proportion of minor, intermediate and major cases to that carried out during the study period.

Lastly, the surgical wrap supplier was contacted regarding the carbon footprint of their surgical wrap. Unfortunately, they were unable to provide this information. A literature search was then conducted to identify the carbon cost of producing polypropylene from oil and the carbon cost of producing surgical wrap from polypropylene. The annual CO_2_ equivalent of polypropylene wrap used for all gynaecology cases performed in 2019 was then estimated.

## Results

The Gynaecology Theatre 1 at CUMH facilitated 66 surgical cases using an estimated 156 surgical sets over 17 days of surgery between 24/1/22 and 1/3/22. Minor surgeries accounted for 53% of cases, intermediate surgeries for 17% and major surgeries for 30% of cases. (Table 1). Average number of surgical sets, average number of sheets of wrap and average area of surgical wrap for minor, intermediate and major surgeries are shown in Table 2.

**Table 1:** Surgeries performed and surgical instrument set use from 24/1/22 to 1/3/22

**Procedure type**	**Typical number of instrument sets used per procedure**	**Number of procedures carried out**	**Estimated number of instrument sets used**
**Minor**			
Cystoscopy	2	7	14
Hysteroscopy D + C	2	19	38
Incision & drainage of Bartholin’s abscess	1	2	2
Vulval/vaginal biopsy	1	5	5
Cone/LLETZ biopsy	2	1	2
Endometrial ablation	2	1	2
		**35 (53% of cases)**	**63**
**Intermediate**			
Operative laparoscopy (tubal sterilisation or USO/BSO)	4	5	20
Pelvic floor repair	3	4	12
Partial vulvectomy	1	2	2
		**11 (17% of cases)**	**34**
**Major**			
Radical vulvectomy	3	1	3
Total abdominal hysterectomy ± BSO ± debulking ± omentectomy	2	12	24
Laparotomy ± USO/BSO (staging)	2	1	2
Laparoscopic hysterectomy ± BSO (straight stick / RA)	5	6	30
		**20 (30% of cases)**	**59**
**Total**		**66**	**156**

*D *+ *C* dilatation and curettage, *LLETZ* large loop excision of transformation zone, *USO/BSO* unilateral salpingo-oophorectomy/bilateral salpingo-oophorectomy, *RA* robot assisted

**Table 2:** Surgical wrap data

**Estimated number of instrument sets used during study period**	156		
**Estimated number of surgical wrap sheets potentially collectable/recyclable (2 sheets of wrap per set)**	312		
**Actual number of wrap sheets collected**	221 (71% of estimated)		
**Actual surface area of wrap sheets collected**	282.1 m^2^		
**Average area of each wrap sheet**	1.28 m^2^		
**Average surgical wrap use per case**	**Sets**	**Sheets**	**Area**
*Minor*	1.8	3.6	4.61m^2^
*Intermediate*	3.1	6.2	7.94m^2^
*Major*	2.9	5.9	7.55m^2^

In total, 221 individual sheets of surgical wrap were collected. The average area per sheet of wrap was 1.28 m^2^. This equated to a surface area of 282.1 m^2^ of surgical wrap collected (Table 2). To provide an easy visual representation, it was calculated that the amount of surgical wrap diverted to the recycling stream would cover approximately one and a half (1.44) International Tennis Federation-sized singles tennis courts [6]. Information provided by the manufacturer stated that the green and blue layers of surgical wrap had different densities. The average density of the surgical wrap was calculated as 61.5 g/m^2^. The total weight of surgical wrap recycled was 17.35 kg.

The findings of the pilot study were extrapolated to estimate the amount of surgical wrap used in public sector gynaecology surgery cases in Cork and the carbon emissions associated with this amount of surgical wrap on an annual basis. In 2019, the most recent year during which operating theatres ran normally prior to the COVID-19 pandemic, 1909 gynaecological surgeries were performed [7]. Based on the same proportions of minor, intermediate and major surgeries as were carried out during the study period, it was calculated that 711.2 kg of surgical wrap could be recycled annually. This equates to 11,564 m^2^, an area covered by 59 tennis courts.

Using information provided by the CUMH waste officer regarding waste processing costs, it was estimated that disposal of 711.2 kg of polypropylene surgical wrap in the general waste stream would cost €107 per annum. Disposal of the same quantity of wrap in the recycling stream would cost approximately €35 per annum. The waste management company does not invoice the hospital for recycling of the surgical wrap on the basis that CUMH are renting a baler from them at an annual cost of €840. Thus, ongoing recycling of an unlimited amount of surgical wrap will come at an annual cost of €733.

While it was not possible to obtain a precise value for the carbon footprint of the surgical wrap from the manufacturer, a useful publication by the UK Department for Business, Energy and Industrial Strategy calculated the carbon cost of producing “formed” polypropylene from oil to be 3.11 kCO_2_ eq./kg [8]. Whilst not specific to surgical wrap, and until the manufacturer completes carbon emissions analysis of their product, it is the closest calculation that could be found. Transport costs also incur an additional carbon spend. The manufacturer calculated that blue surgical wrap travels 17,605 kms from its manufacturing and processing site in China to Dublin, while green surgical wrap travels 11,705 kms from India to Dublin. The additional carbon spends incurred in this transport chain is unknown and this information is currently unavailable from the manufacturer. Thus, an estimated carbon emissions equivalent of at least 53.95 kg was associated with the surgical wrap collected during the study period, and at least 2.2 tonnes of CO_2_ Eq are associated with the amount of surgical wrap used for gynaecological surgeries in Cork annually.

## Discussion

This study demonstrates that the implementation of a simple operating theatre recycling initiative can reduce the carbon footprint of our hospitals. As a result of this pilot study, all surgical wrap from both gynaecology theatres at CUMH is now being recycled. The wrap is baled in the hospital waste yard and the polypropylene bales are collected by the hospital waste management company on a weekly basis. The immediate goal is to roll this recycling initiative out to all four operating theatres in CUMH.

**Fig. 2:**
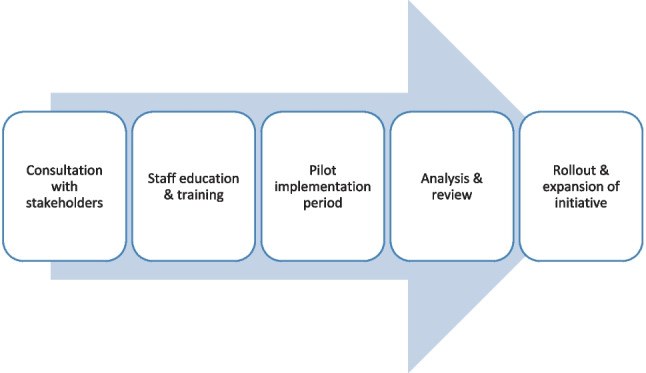
Template for introduction of a healthcare recycling initiative

This pilot project has created a healthcare recycling template (Fig. 2) which has stimulated a similar initiative in CUMH’s co-located hospital, Cork University Hospital. Additionally, expressions of interest have been received regarding implementation of this initiative in a Dublin hospital. It is hoped that, through hospital regional sustainability officers, similar initiatives will be rolled out regionally and nationally. To achieve this goal, traditional barriers to healthcare sustainability will need to be overcome. These include a lack of environmental awareness and education among hospital staff, a poor recycling culture and “numbness” regarding environmental issues, endemic in the healthcare sector, often due to inadequate staffing and resourcing. Hopefully, with an increased emphasis recently on carbon budgets across all sectors of society, both staff and hospital management across the country will come to a greater appreciation of the urgency and importance of such changes in practice for the benefit of our hospitals and our planet.

Engagement with the relevant stakeholders was crucial to the success of this study. Fortunately, there was significant buy-in from theatre management, nursing staff, theatre and waste porters, and the hospital waste management company. The project faced a number of challenges. It took time to translate this idea into practice while staff were educated and while the logistics were teased out with theatre staff and the waste management company. There were initial cost and storage implications: a unique storage container had to be identified for the surgical wrap in the theatre set-up room and assurances had to be obtained from staff that no other waste would be disposed of there. It was necessary to purchase a large wheelie-bin for the theatre waste room to store the surgical wrap collected daily from theatre during the study period. It was also necessary to rent and store a baler in the hospital waste yard specifically for baling the surgical wrap.

Based on the estimated number of surgical sets used during the study period, it was calculated that 71% of surgical wrap sheets were collected for recycling. The cause of this discrepancy is unclear. A limitation of this study is that the number of sets used for each surgical case were not prospectively documented, so it is likely that fewer surgical sets were used than estimated. It may also be that in the initial days of the project some of the surgical wrap was disposed of in the general waste stream. As the recycling practice becomes embedded in the gynaecology operating theatre, it is anticipated that disposal in the appropriate waste stream will be routine practice.

It is recognised that recycling of surgical wrap is not the only potential option for improving sustainability with regard to surgical sets. Other opportunities include reducing or eliminating the need for surgical wrap created from petrochemicals in the theatre setting. Replacing surgical wrap completely by using rigid sterilisable containers (RSCs) is a lower-carbon option for operating theatres. This approach, though involving significant capital outlay for the purchase of the sterilisation containers, still well outperforms recycling of surgical wrap from an environmental perspective and is thus a more sustainable practice [9]. Returning to the use of reusable fabric surgical wrap could also be considered. Close collaboration with the hospital sterile services department would be necessary in planning these changes. Another approach is to reduce the number of individual sets required for surgical cases. On the basis of this study, consideration is being given to merging some surgical sets at CUMH. The aim is to create more procedure-specific sets for commonly performed surgeries so as to reduce the number of individual surgical sets being used for these cases.

While the recycling of surgical wrap at CUMH is not currently a cost-neutral process due to the need to rent a baler, the acquisition of funding for the purchase of a baler is being explored through the hospital regional sustainability officer. There is also a possibility of purchasing a polypropylene processer which would allow the partial recycling of the wrap on-site. These processes will be more cost-effective once larger volumes of surgical wrap from both co-located hospitals are being diverted to the recycling stream.

Diversion of surgical wrap waste from the general or clinical waste streams to the recycling stream is something that is achievable, with some planning, in every operating theatre. This study has shown that small changes to operating theatre waste disposal practices have the potential to yield reductions in general waste outputs and carbon emissions in a healthcare setting, as well as contributing to the circular economy. It is hoped that this pilot project will spur other areas of healthcare to look at their current waste disposal practices and initiate similar environmentally minded projects.
